# Micro-Biomechanics of the Kebara 2 Hyoid and Its Implications for Speech in Neanderthals

**DOI:** 10.1371/journal.pone.0082261

**Published:** 2013-12-18

**Authors:** Ruggero D’Anastasio, Stephen Wroe, Claudio Tuniz, Lucia Mancini, Deneb T. Cesana, Diego Dreossi, Mayoorendra Ravichandiran, Marie Attard, William C. H. Parr, Anne Agur, Luigi Capasso

**Affiliations:** 1 University Museum – State University “G. d’Annunzio”, Piazza Trento e Trieste 1, Chieti, Italy; 2 Computational Biomechanics Research Group, Zoology, School of Environmental and Rural Sciences, University of New England, New South Wales, Australia; 3 Multidisciplinary Laboratory, The “Abdus Salam” International Centre for Theoretical Physics, Strada Costiera 11, Trieste, Italy; 4 Centre for Archaeological Science, University of Wollongong, Wollongong, New South Wales, Australia; 5 Elettra-Sincrotrone Trieste, Area Science Park, Basovizza, Trieste, Italy; 6 Division of Anatomy, University of Toronto, Department of Surgery, Toronto, Ontario, Canada; 7 Computational Biomechanics Research Group, School of Biological, Earth and Environmental Sciences, University of New South Wales, Kensington, New South Wales, Australia; University of Kansas, United States of America

## Abstract

The description of a Neanderthal hyoid from Kebara Cave (Israel) in 1989 fuelled scientific debate on the evolution of speech and complex language. Gross anatomy of the Kebara 2 hyoid differs little from that of modern humans. However, whether *Homo neanderthalensis* could use speech or complex language remains controversial. Similarity in overall shape does not necessarily demonstrate that the Kebara 2 hyoid was used in the same way as that of *Homo sapiens*. The mechanical performance of whole bones is partly controlled by internal trabecular geometries, regulated by bone-remodelling in response to the forces applied. Here we show that the Neanderthal and modern human hyoids also present very similar internal architectures and micro-biomechanical behaviours. Our study incorporates detailed analysis of histology, meticulous reconstruction of musculature, and computational biomechanical analysis with models incorporating internal micro-geometry. Because internal architecture reflects the loadings to which a bone is routinely subjected, our findings are consistent with a capacity for speech in the Neanderthals.

## Introduction

The Kebara 2 Neanderthal dates from approximately 60 ka and is part of a near-complete adult male skeleton unearthed in 1983 [1]. Subsequent discoveries of additional fossil hominin hyoids have generated renewed interest in the bone’s potential to inform on the evolution of speech and complex language. These include: a partial Neanderthal hyoid (SDR-034) from El Sidròn Cave (Asturias, Spain) dated to ∼43 ka [2]; two Middle Pleistocene hyoids (AT-1500 and AT-2000) assigned to *Homo heidelbergensis* from Sierra de Atapuerca (Spain) dated at ∼530 ka [3]; and a “chimpanzee-like” hyoid assigned to *Australopithecus afarensis* from Dikika (Ethiopia, ∼3.3 Ma) [4].

**Figure 1:**
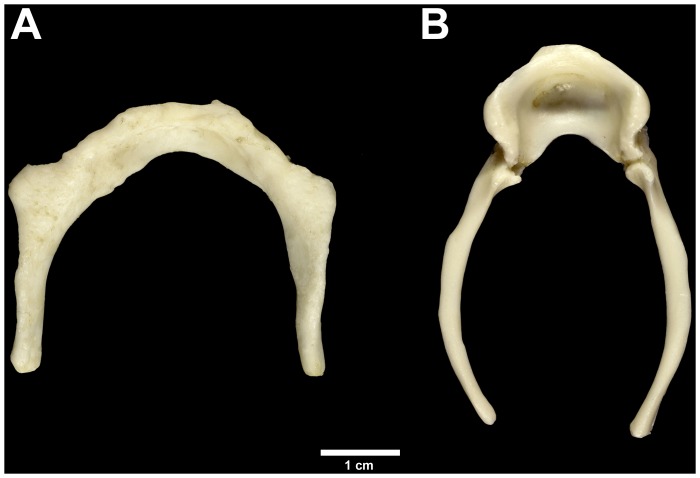
Male *Homo sapiens* and *Pan troglodytes* hyoid bones. Note that the human hyoid (A) lacks the large and distinctive bulla of the chimpanzee hyoid (B). Specimens are research quality casts numbers 844 and 837 held at the University Museum, Trieste.

**Figure 2:**
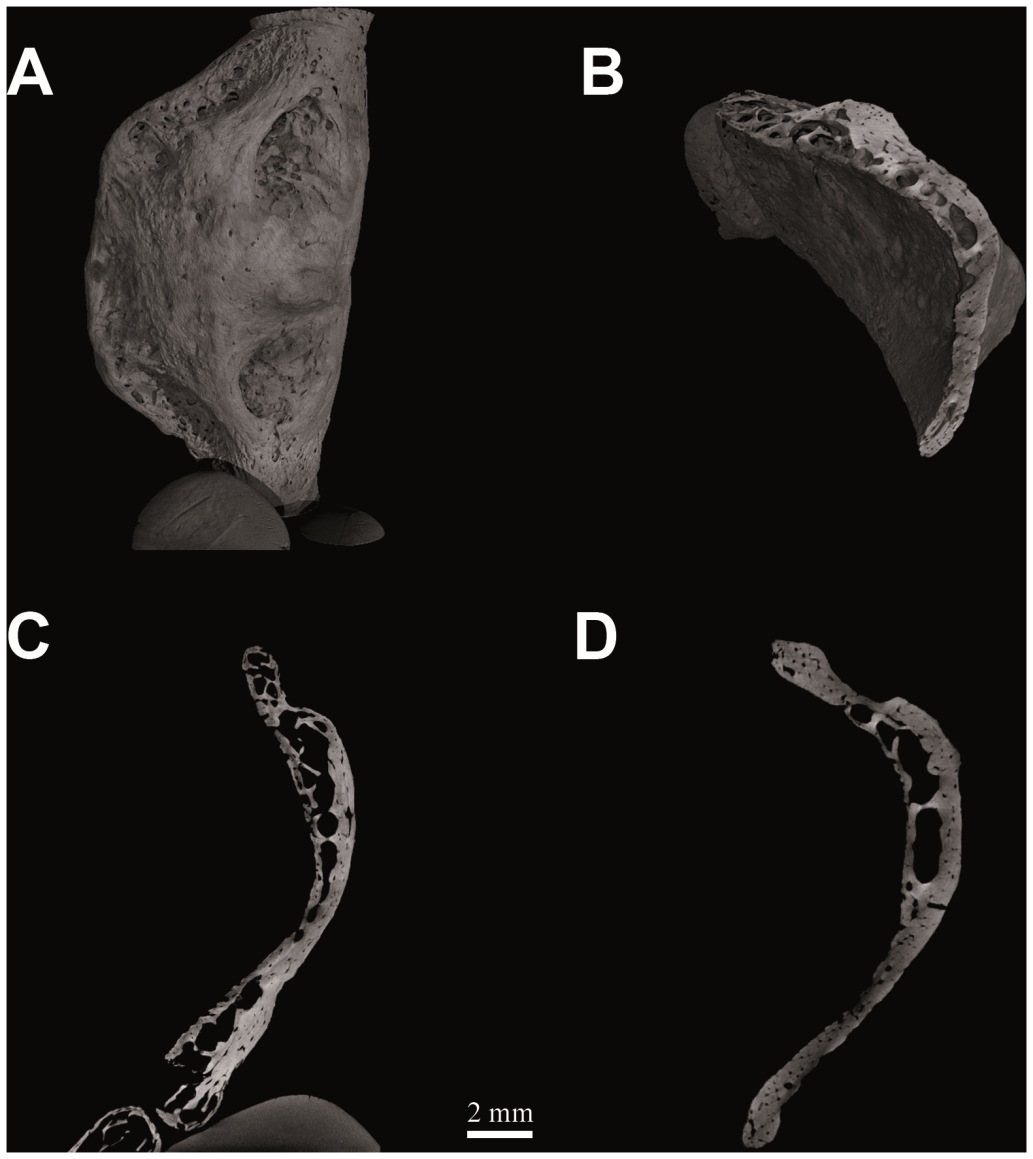
Figure 2. Computed tomography of *Homo neanderthalensis* (Kebara 2, Tel Aviv University - Israel). Hyoid body volume rendering (V = 80 kV, I = 100 µA; pixel size: 10.0 µm; exposure time: 3.0 sec.; 2400 projections over 360 degrees) (a); spongy bone structure (b); histological architecture: medial sagittal section (c) and medial transverse section (d).

Gross anatomy of the hyoid in *Pan troglodytes*, which includes a cup-shaped extension or bulla (also present for the Dikika *A. afarensis* specimen), is very different to that of modern humans (Figure 1). However, analyses of gross macroscopic anatomy in the Kebara 2 hyoid (Figure 2A), as well as SDR-034, have shown that the hyoid of *H. neanderthalensis* was almost indistinguishable from that of modern humans [1], [2]. Similarly, anatomical and anthropometric descriptions of the Sima de los Huesos material show that the hyoid of *H. heidelbergensis* was modern-human-like [3]. Thus, it appears that the external macroscopic morphology of this important component of the vocal apparatus in modern humans had arisen by ∼530 ka and has remained largely unchanged since.

Overall similarity between the external morphology of the Kebara 2 hyoid and those of modern humans has suggested to some researchers that the Kebara 2 Neanderthal was capable of speech, and perhaps language [1], [5]. Others have contested this conclusion, and, whether or not Neanderthals could speak remains a contentious issue [6]–[8]. Certainly a bone’s overall shape and external dimensions alone provide incomplete understanding of its precise function [9]. More detailed and specific insights into mechanical performance are reflected in the geometry of internal microstructure, including trabecular networks that are controlled through bone remodelling [9]–[11]. In remodelling, bone is resorbed or new ossification takes place, largely in response to mechanical loading. This is manifested in the specific morphology and orientation of the bony trabeculae and the size and distributions of the osteons [12], [13]. As observed in other fossil bones, histological structure reflects the forces imposed by muscles [14] and sound-waves during phonation [15].

If the hyoid body of Kebara 2 was being used in a different way from those of modern humans then we would expect to observe clear differences in its histology and micro-biomechanical behaviour. In the present study we ask whether this is so. Based on microCT data [16], our analyses include comparisons of histological structure and micro-biomechanical performance applying Finite Element Analysis (FEA) of high-resolution models that incorporate trabecular network geometry.

FEA is a powerful engineering tool originally developed for the aerospace industry to enable the non-destructive prediction of mechanical behaviour in man-made structures. Predicting mechanical behaviour in complex shapes using traditional analytical approaches is problematic. Unlike analytical methods, where exact solutions to partial differential equations are sought, FEA is a computational technique that converts the problem into a system of multiple simultaneous algebraic equations for simple shapes, solutions to which yield approximate values of the unknowns at a discrete (finite) number of points in the continuum. This process of modeling an object by dividing it into a system of smaller elements of known geometry (finite elements), interconnected at points common to two or more elements (nodes), is called discretisation. FEA is now increasingly used in biology [17], biomedicine, palaeontology and physical anthropology [18]–[20].

It is important to note that the modelling approach used in the present study is entirely comparative, as in previous broadly similar studies [18]–[20]. We stress that it is not our objective here to predict material failure or absolute values for indicators of mechanical performance. In this context the actual material properties for bone are largely unimportant, because there is no compelling reason to believe that there are major differences in these properties between modern humans and Neanderthals. The objective is to determine any differences in a relative context.

Only two studies to date have analysed micro-biomechanical models that capture the trabecular network geometry of whole bones [9], [21], and none known to us has been performed on a whole fossil bone of any taxon. The loadings applied to our models are based on very detailed 3D reconstruction of hyoid musculature to the level of fiber bundles. Models were scaled to account for size differences and subjected to identical loadings based on data from the muscle reconstruction.

## Results and Discussion

**Figure 3:**
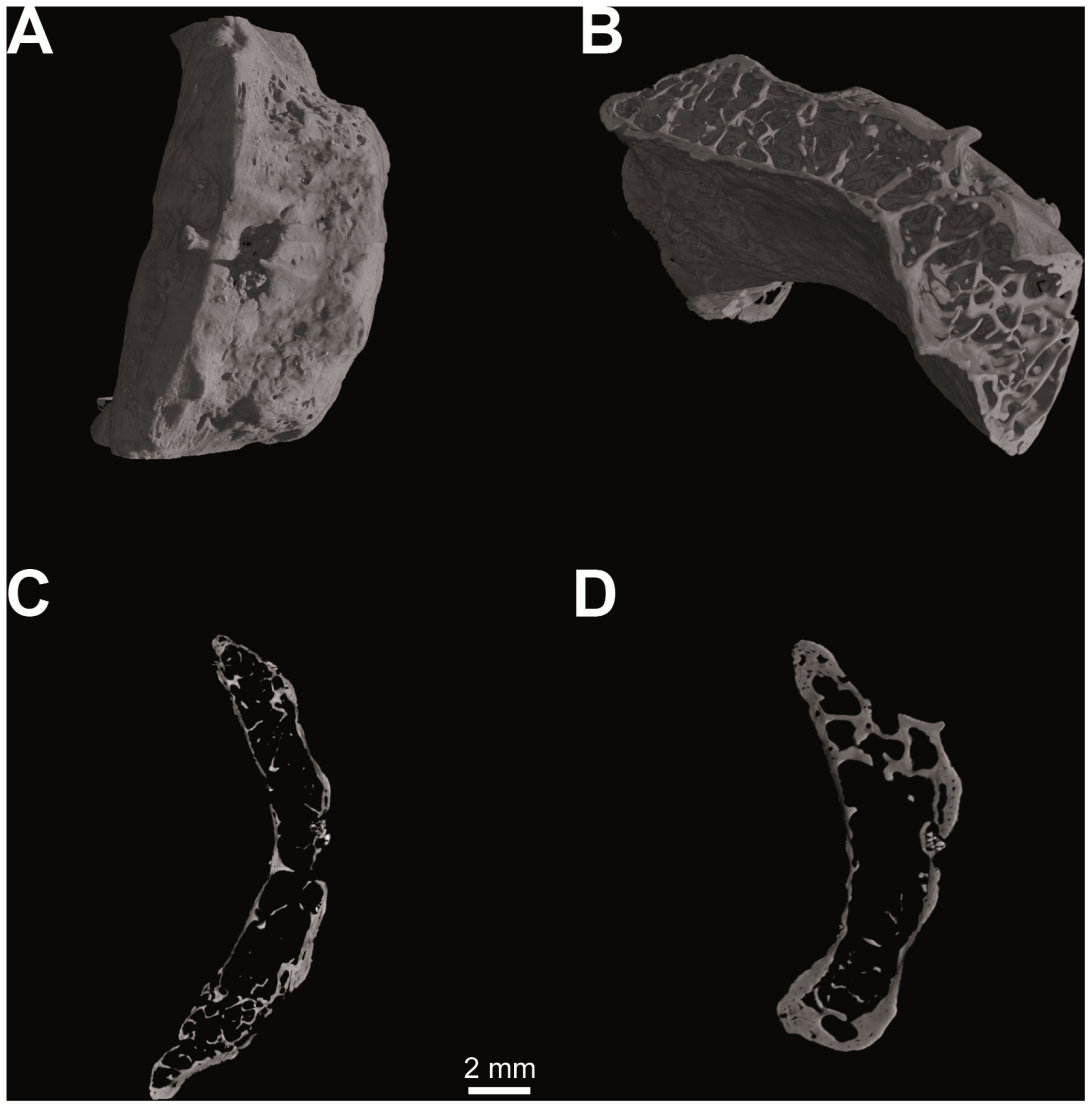
Computed tomography of *Homo sapiens* (N. S36-Sulmona Fonte d′Amore T64, University Museum Chieti - Italy). Hyoid body volume rendering (V = 80 kV, I = 100 µA; pixel size: 12.5 µm; exposure time: 2.0 sec.; 2400 projections over 360 degrees) (a); spongy bone structure (b); histological architecture: medial sagittal section (c) and medial transverse section (d).

Examination of internal microscopic anatomy reveals that the medial sagittal microCT section from the Kebara 2 hyoid body (Figure 2) shows a marked arcuate shape, corresponding with deep fossae for the insertion of the geniohyoid muscles [1]. Its histomorphology (Figure 2) is characterized by cortical bone with vascular channels, well-developed intertrabecular spaces and dorsoventrally oriented bony lamellae. In each of these respects its microarchitecture is comparable to that of modern human hyoids (Figure 3).

**Figure 4:**
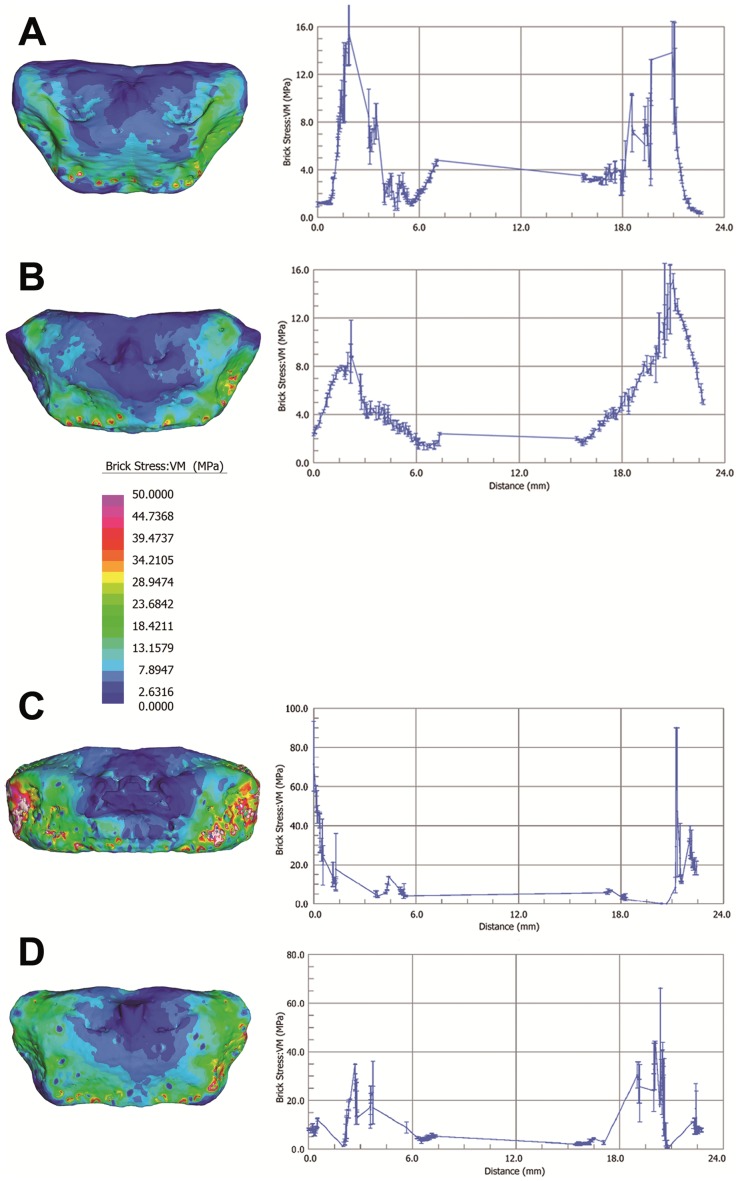
Computational biomechanical analyses of hyoid models. *Homo neanderthalensis* (Kebara 2) (A), *Homo sapiens* (SAT37) (B), *Homo sapiens* (OP1T37) (C), and *Homo sapiens* (SAT41) (D). Surface von Mises stress distributions in visual plots for each model are given for each model on the left. On the right a two dimensional graph, generated using the Graph Tool (Vs Position) in Strand7 (2.4), is provided. This gives von Mises stress for internal elements intersecting a straight line drawn between nodes at maximum lateral width of each hyoid body, i.e., between the lateral most extremes where the body would have connected with the hyoid’s greater cornua (greater horns). Values are interpolated across element edges intersected by the line. MPa = megapascals.

Visual plots of von Mises (VM) stress distributions are given in Figure 4. VM stress is a good indicator of material failure in relatively ductile materials such as bone [22]. Mean values for VM stresses are given for each Finite Element Model (FEM) as a whole and for subgroups containing only surface elements in Table 1. Using the Graph Tool in Strand7 (2.4) a straight-line was drawn between the dorso-lateral-most extremes of each model to plot a graph of VM stress for elements intersecting the line (Figure 4).

**Table 1:** Mean element Von Mises stresses.

VM stress (MPa)	Kebara	SAT37	OP1T37	SAT41
Mean	6.3	6.4	8.9	10.8
SD	5.31	5.9	10.7	13.9
Mean for surface elements	8.3	8.3	12.6	13.1
SD Mean for surface elements	6.9	7.3	14.7	16.1

MPa = megapascals, SD = Standard Deviation.

Results from visual plots, mean overall, mean surface and straight-line VM stress values all suggest that in terms of both VM stress magnitudes and distributions, both internally and externally, the mechanical behavior of the Kebara 2 hyoid is very similar to that of hyoids from male modern humans under identical loading. Although, on the basis of mean element VM stress, the *H. neanderthalensis* specimen (6.3 MPa) falls just outside the range determined for the *H. sapiens* sample (6.4 MPa to 10.8 MPa), it is in fact both qualitatively and quantitatively much closer to SAT37 than this modern human hyoid is to the remaining two (Figure 4, and Table 1).

MicroCT analysis reveals that the hyoid bodies of both Kebara 2 and modern humans are characterized by two thick cortical layers, well-defined vascular channels and well-developed spongy structures. The detail of histological structure in all specimens, including Kebara 2, is typical of bone involved in intense and continuous metabolic activity. Our analysis shows that the similarity in gross surface morphology between the Kebara 2 hyoid and those of modern humans also extends internally to microscopic architecture and the orientations of the bony trabeculae comprising the spongy bone of the hyoid body.

The results of FEA-based comparisons of our high-resolution models further show that the Kebara 2 hyoid presents very similar micro-biomechanical performance to that of modern humans under identical loadings. Minor histomorphological differences are present in that the Kebara 2 hyoid appears more dorsoventrally flattened in the distal regions, and the bony trabeculae appear thicker than in our modern human sample. However, given the considerable variation among the modern human specimens we consider it likely that these differences are a manifestation of individual histological variability and/or size differences.

The hyoid undoubtedly plays an active role in speech and is indicative of the state of the vocal tract. As the vocal tract’s only ossified element, it is the only part likely to be preserved in the fossil record. It is not directly attached to any other bone in the skeleton, being held by ligaments and muscles that attach it to the mandible, temporal bone, thyroid cartilage and sternum. It provides support for the larynx and anchorage for the tongue and other muscles required for speaking. However, other muscles not attached to the hyoid are important in human speech, which is ultimately under neurological control.

In sound production, the tongue assumes configurations that influence the morphology of the vocal tract largely in response to contractions of its intrinsic muscles [23]. However, changes in overall tongue position relative to the hard palate are the result of hyoid movements controlled by differential activity in the hyoid and extrinsic tongue muscles [24], [25].

Although the hyoid moves continuously during speech its movements are not linked to jaw movement. A clear dichotomy has been identified in hyoid movement patterns generated during feeding and those generated during speech. These different behaviours of the hyoid are marked by a shift in the operating length of the anterior and posterior suprahyoid muscles, such that the anterior group (especially the geniohyoid) are functionally ‘shorter’ and the posterior group functionally ‘longer’ in speech than in feeding [26], [27]. This confirms earlier work suggesting that activation patterns in the mandibular muscles during speech are not related to the rhythmic patterns of chewing [28].

Modern-human-like gross anatomy in the hyoid body of a fossil specimen is not, in itself, clear demonstration that the individual was capable of speech [6]. However, our analyses demonstrate that previously observed gross similarities between the Kebara 2 hyoid and those of modern humans are not superficial.

We conclude that the presence of modern-human-like histological features and micro-biomechanical behavior in the Kebara 2 hyoid indicates that this bone not only resembled that of a modern human, but that it was used in very similar ways. This is because the internal microarchitecture is a response to the vectors and magnitudes of the forces to which it is routinely subjected. These findings are consistent with the suggestion that the Kebara 2 Neanderthal practiced speech (*sensu* Duchin 1990) [29] although they do not prove that this was so. We are also mindful of the fact that our sample size is small and that the addition of further models of more modern human material, as well as specimens of *Pan troglodytes* and/or *Pan paniscus*, are needed before any firmer conclusions could be drawn.

Previous studies have shown that anatomical features of the outer and middle ear associated with the perception of speech were also present in *H. heidelbergensis* [30]. Based on recent vocal tract reconstruction of both *H. heidelbergensis* (cranium 5 from Sima de los Huesos) and *H. neanderthalensis* (La Ferrassie 1) and comparisons with modern humans, it has been inferred that not only *H. neanderthalensis*, but perhaps this common ancestor of both Neanderthals and modern humans may have been capable of speech [31]. Micro-biomechanical modeling of hyoid material referred to *H. heidelbergensis* could help to resolve this question.

Given that our results add support for the proposition that the Kebara 2 Neanderthal engaged in speech, the question may then become was he capable of the critical thought and syntactical ability necessary for complex language? Conclusive resolution of this question is not possible with the data and analytical tools currently available. However, speculation on this issue might be considered in light of the mounting body of evidence that continues to expand the known repertoire of sophisticated subsistence strategies and symbolism practiced by Neanderthals [32]–[37].

## Materials and Methods

The Kebara 2 Neanderthal hyoid is stored in the Department of Anatomy and Anthropology of the Sackler Faculty of Medicine, Tel Aviv University, Tel Aviv, Israel. The modern human samples (N. S1365-SAT37; N. S1363-SAT41; N. S548-OPIT37) are stored in the University Museum of the University “G. d’Annunizio”, Chieti, Italy. These are all thought to be male, as is Kebara 2, with estimated ages of 20–40, 40–60 and 35–40 years respectively.

Microfocus X-ray microCT was performed at the TomoLab station, Elettra Synchrotron Light Laboratory, Trieste Italy. Volumes of the whole hyoid samples were reconstructed from tomographic projections acquired through sample rotations over 360 degrees (Kebara 2 = 2400 projections, voxel size 10 microns; modern hyoid samples = 1800 projections, voxels size 18 microns). Volume analysis was carried out both on the reconstructed 2D slices and on the rendered volumes. Volume renderings were obtained using VGStudio MAX 2.0©.

**Figure 5:**
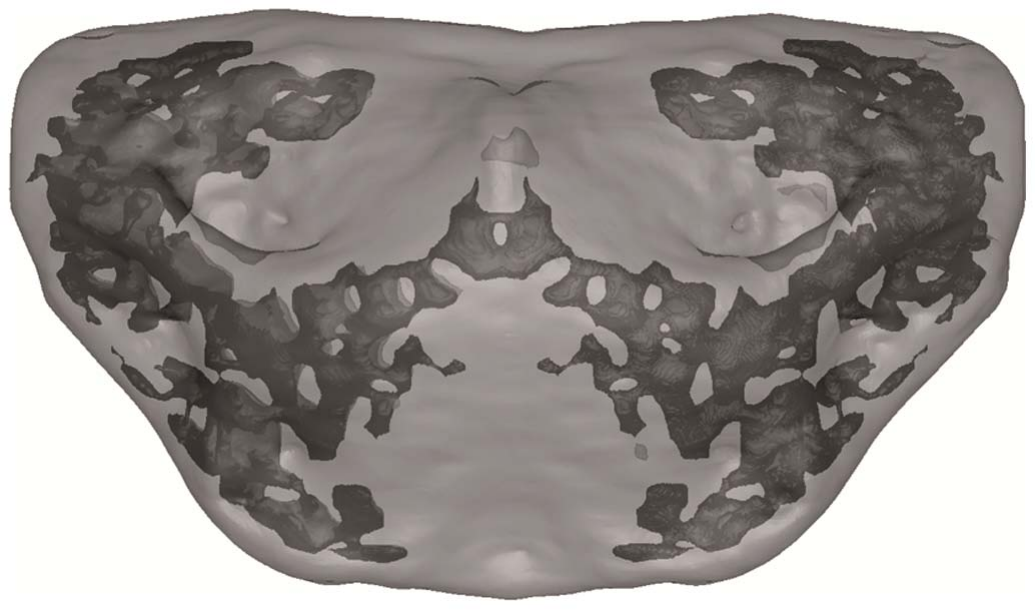
Transparent image of Finite Element Model of the Kebara 2 hyoid. Porous internal structure highlighted in dark grey.

‘Porous’ three dimensional Finite Element Models capturing the cortical bone and trabecular networks were created in MIMICS 13.4 for the Kebara hyoid and those of the three modern humans (Figures 4 and 5). Previous modeling of whole human bones that incorporates the porous internal structure, i.e., the geometry of trabecular networks, has indicated that this generates more accurate results than can be achieved using non-porous solid models, or models that attempt to approximate the properties of trabecular bone based on CT density data [9].

FEMs for the Kebara 2 hyoid and those of SAT37, OPIT37, and SAT41 comprised 1,482,720, 1,512,145, 1,526,070 and 1,496,338 4-noded tetrahedral ‘brick’ elements respectively. Specific material properties for the human hyoid are unknown. In this study, elements were designated as isotropic and assigned a Young’s modulus of 13 GPa and Poisson’s ratio of 0.3 following previously published methods [10]. We reiterate that, in the comparative context in which our approach is applied, actual material properties are unimportant unless major differences in properties are thought to exist between specimens and stress magnitudes should be interpreted as relative and not absolutes values [11], [17].

**Figure 6:**
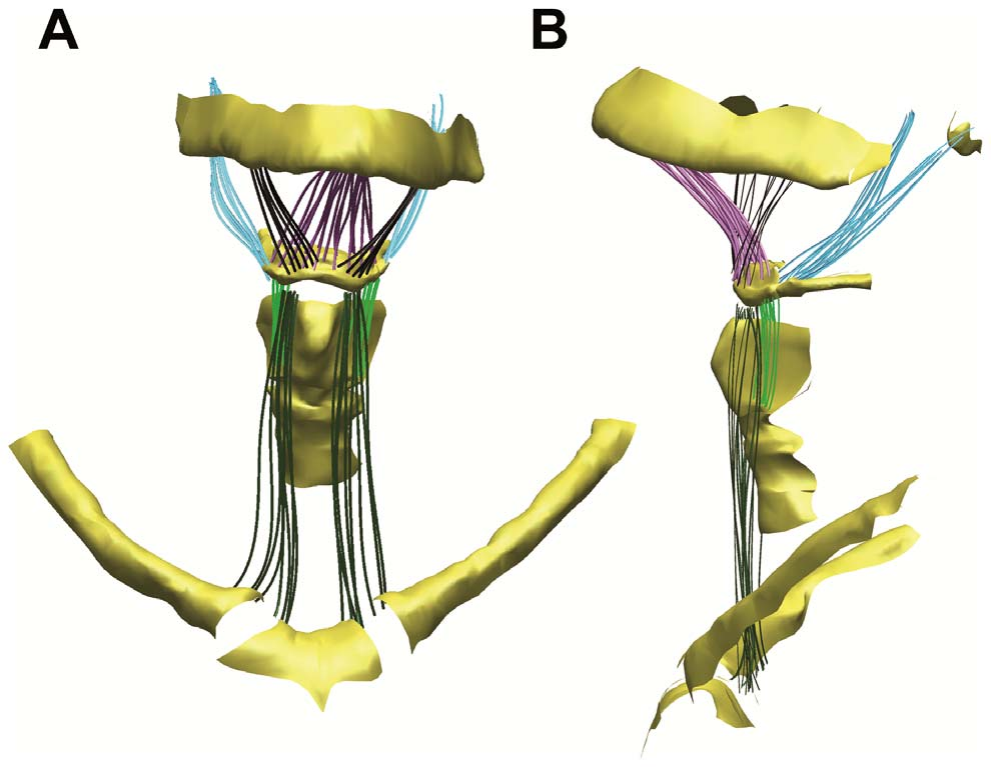
3D reconstruction of hyoid musculature to the level of fiber bundles. Muscles of the human hyoid used to determine forces and vectors applied in Finite Element Analyses reconstructed in 3D. Frontal view (a) and lateral view (b). Geniohyoid (purple); Mylohyoid (black); Stylohyoid (blue); Sternohyoid (green).

Forces and vectors were calculated for individual muscle fibers based on detailed 3D reconstruction (Figure 6). The sternohyoid, stylohyoid, geniohyoid, thyrohyoid and mylohyoid muscles, along with their bony attachments and trachea, were serially dissected and digitized using a Microscribe 3DX digitizer. The coordinate data was imported into Autodesk™ Maya™ 2012 and reconstructed into a 3D model. Custom software was used to calculate the physiological cross-sectional area and volume for each of the muscles [38] (and see Table 2 and Figure 6).

**Table 2:** Hyoid muscle physical cross-sectional areas and forces.

Muscle	PCSA (mm^2^)	Muscle force (N)	No. of fibers
Sternohyoid [R]	83.27	24.98	6
Sternohyoid [L]	74.25	22.27	6
Geniohyoid	252.40	75.72	12
Mylohyoid [L]	135.90	40.77	6
Mylohyoid [R]	173.45	52.03	6
Stylohyoid [L]	23.38	7.01	6
Stylohyoid [R]	28.26	8.48	6
Thyrohyoid [L]	52.06	15.62	6
Thyrohyoid [R]	60.77	18.23	6

All Finite Element Analyses were performed in Strand7 (2.4) using a Dell Precision T1500 (64 bit, Core i7, 16.0 GB RAM). Finite Element Models were scaled [11] to the same maximal width of 24 mm determined for the specimen from which muscle data was collected. In order to minimize the appearance of artifacts that can be generated by point loadings, forces were applied to nodes embedded within networks of fine beams tessellated into the models’ surfaces [39]. Models were fixed in translation but left free in rotation at two points on each of the synovial joints about the long axis. All analyses were linear-static.
